# Arylglycine-derivative synthesis via oxidative sp^3^ C–H functionalization of α-amino esters

**DOI:** 10.3762/bjoc.8.178

**Published:** 2012-09-18

**Authors:** Zhanwei Xu, Xiaoqiang Yu, Xiujuan Feng, Ming Bao

**Affiliations:** 1 State Key Laboratory of Fine Chemicals, Dalian University of Technology, Dalian 116023, China

**Keywords:** α-amino ester, arylglycine, C–H functionalization, oxidation, synthesis

## Abstract

An efficient method for the synthesis of arylglycine derivatives is described. The oxidative coupling reactions of naphthols and phenols with α-amino esters proceeded smoothly in the presence of *meta*-chloroperoxybenzoic acid as an oxidant under ambient conditions, to produce arylglycine derivatives in satisfactory yields.

## Findings

**Scheme 1:**
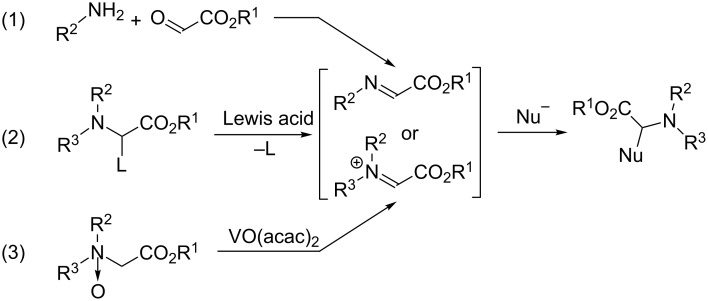
Synthesis of arylglycine derivatives.

Arylglycine derivatives represent important synthetic intermediates or building blocks for drug development and natural-product synthesis [1–2]. The arylglycine moiety also occurs in several bioactive natural products [3]. Consequently, the development of convenient and efficient methods for the preparation of arylglycine derivatives has attracted considerable attention. Over the past years, many methods have been developed for the preparation of arylglycine derivatives [3]. Among these, the addition reaction of a carbon nucleophile to imines or iminium ions through Mannich-type reaction appears more useful (Scheme 1, reactions 1–3). However, these reactions need expensive arylboronic acids (Petasis reaction) [4–9] and suitable leaving groups [10–12] as well as a metal catalyst (Polonovsky reaction; this route requires the preparation of amine *N*-oxide in advance) [13–14].

**Scheme 2:**
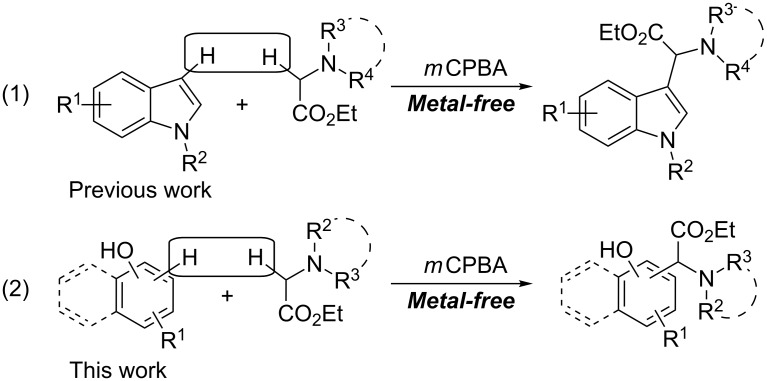
Oxidative sp^3^ C–H functionalization of α-amino esters.

We have recently reported the copper-catalyzed oxidative coupling reaction of alkynes with tertiary amine *N*-oxides [15]. This new strategy for the direct functionalization of sp^3^ C–H bonds adjacent to a nitrogen atom, via tertiary amine *N*-oxide intermediates, was successfully applied to the coupling reaction of ethyl 2-(disubstituted amino)acetates with indoles to achieve indolylglycine derivatives (Scheme 2, reaction 1) [16]. In the course of our continuous research on the direct functionalization of sp^3^ C–H bonds, we found that this new strategy could also be applied to the coupling reaction of naphthols and phenols with ethyl 2-(disubstituted amino)acetates. The results are reported in the current work (Scheme 2, reaction 2).

In our initial studies, the reaction of 2-naphthol (**1a**) with ethyl 2-morpholinoacetate (**2a**) was chosen as a model for optimizing the reaction conditions. The results are shown in Table 1. The proportions of substrate **2a** and oxidant *meta*-chloroperoxybenzoic acid (*m*CPBA) were initially screened with CH_3_CN as the solvent (Table 1, entries 1–3). The yield of **3a** was increased to 77% when 1.2 equiv of **2a** and *m*CPBA were used (Table 1, entry 2). Further increasing the amounts of **2a** and *m*CPBA or adding a copper catalyst could not improve the yield of **3a** (Table 1, entries 3 and 4). The solvents were then screened (Table 1, entries 5–10). The best result was observed when CH_2_Cl_2_ was used as the solvent (79%, Table 1, entry 5). Therefore, the subsequent reactions of naphthols and phenols with ethyl 2-(disubstituted amino)acetates were performed in the presence of *m*CPBA (1.2 equiv) in CH_2_Cl_2_ under ambient conditions.

**Table 1:** Optimization of reaction conditions.^a^

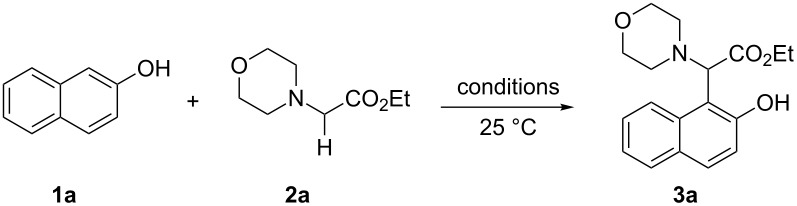					

Entry	**2a** (equiv)	*m*CPBA (equiv)	Time (h)	Solvent	Yield of **3a** (%)^b^

1	1.0	1.0	40	CH_3_CN	63
2	1.2	1.2	40	CH_3_CN	77
3	1.5	1.5	40	CH_3_CN	77
4^c^	1.2	1.2	40	CH_3_CN	75
5	1.2	1.2	24	CH_2_Cl_2_	79
6	1.2	1.2	40	THF	65
7	1.2	1.2	48	dioxane	16
8	1.2	1.2	48	CH_3_CH_2_OH	14
9	1.2	1.2	48	toluene	70
10	1.2	1.2	48	DMF	trace

^a^Reaction conditions: 2-naphthol (**1a**, 72.1 mg, 0.5 mmol), ethyl 2-morpholinoacetate (**2a**, 1.0 equiv to 1.5 equiv), and *m*CPBA (1.0 equiv to 1.5 equiv) in solvent (3.0 mL) under air at 25 °C. ^b^Isolated yield. ^c^10 mol % Cu(OTf)_2_ was used as a catalyst.

The substrate scope was determined under the optimized reaction conditions, and the results are shown in Table 2. As expected, the reactions of ethyl 2-morpholinoacetate (**2a**), ethyl 2-(piperidin-1-yl)acetate (**2b**), and ethyl 2-(benzyl(methyl)amino)acetate (**2c**) proceeded smoothly to give the corresponding products **3a**–**3c** in good yields (Table 2, entries 1–3, 64–79%). These results indicated that both α-cyclic and acyclic amino esters could be employed in this type oxidative coupling reaction. The desired products **3d**–**3f** were obtained in yields of 66–79% from the reactions of naphthols **1b**–**1d** with **2a** (Table 2, entries 4–6). However, relatively low yields were observed from the reactions of phenols **1e**–**1h** with **2a** (Table 2, entries 7–10, 30–55%). The poor reactivity of phenols **1e**–**1h** was considered to be due to their lower electron density compared to naphthols **1b**–**1d**. No reaction was observed from the mixture of phenol **1i**, bearing an electron-withdrawing Br substituent on *para*-position, and **2a** (Table 2, entry 11).

**Table 2:** Oxidative coupling reaction of naphthols and phenols with α-amino esters.^a^

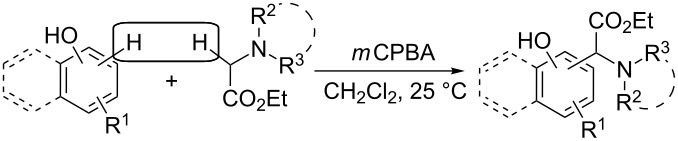					

Entry	Phenol **1**	Amine **2**	Time (h)	Product **3**	Yield (%)^b^

1	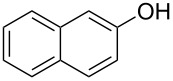 **1a**	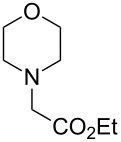 **2a**	24	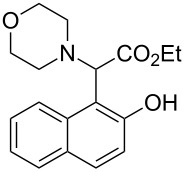 **3a**	79
2	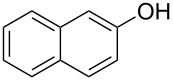 **1a**	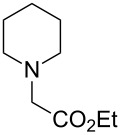 **2b**	24	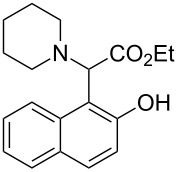 **3b**	64
3	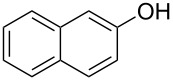 **1a**	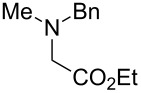 **2c**	36	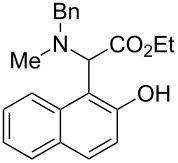 **3c**	64
4	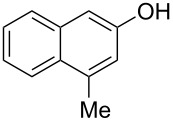 **1b**	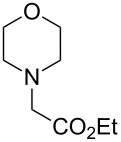 **2a**	20	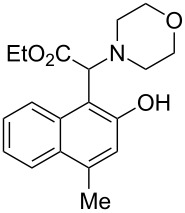 **3d**	79
5	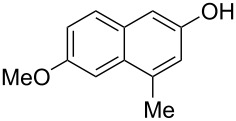 **1c**	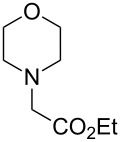 **2a**	20	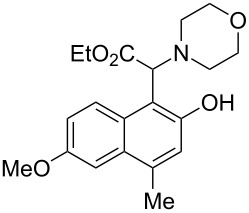 **3e**	75
6	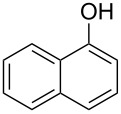 **1d**	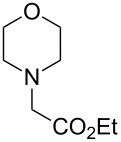 **2a**	18	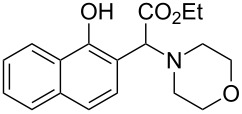 **3f**	66
7	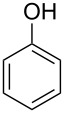 **1e**	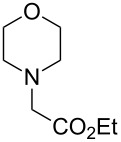 **2a**	48	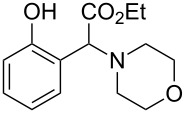 **3g**	30
8	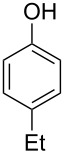 **1f**	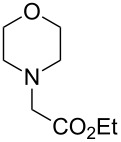 **2a**	36	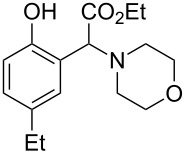 **3h**	30
9	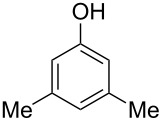 **1g**	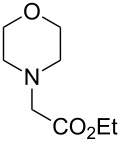 **2a**	24	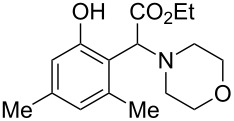 **3i**	35
10	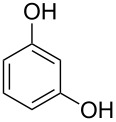 **1h**	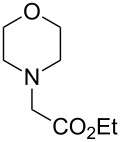 **2a**	16	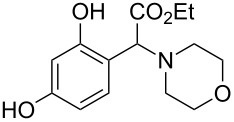 **3j**	55
11	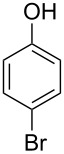 **1i**	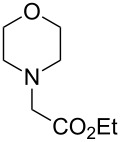 **2a**	48	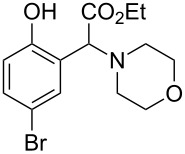 **3k**	0

^a^Reaction conditions: naphthols or phenols (**1**, 0.5 mmol), α-amino esters (**2**, 0.6 mmol, 1.2 equiv), and *m*CPBA (121.8 mg, 0.6 mmol, 85% purity) in CH_2_Cl_2_ (3.0 mL) under air at 25 °C. ^b^Isolated yield.

**Scheme 3:**
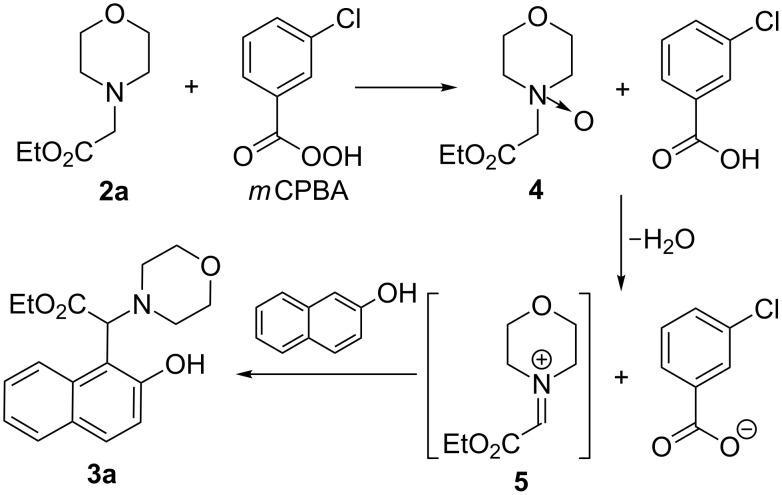
Proposed mechanism.

The plausible mechanism for the coupling reaction of naphthols and phenols with ethyl 2-aminoacetate derivatives is shown in Scheme 3 [16–19]. *m*CPBA oxidized **2a** to amine *N*-oxide **4** before being transformed into 3-chlorobenzoic acid. The interaction of **4** with 3-chlorobenzoic acid led to the generation of the iminium ion **5** and 3-chlorobenzoate anion. The Mannich-type reaction of **5** with 2-naphthol may have occurred to generate the coupling product **3a**. The generated 3-chlorobenzoate anion acted as a proton acceptor.

In conclusion, a new strategy for the functionalization of sp^3^ C–H bonds of amino esters was successfully applied to the coupling reaction of ethyl 2-(disubstituted amino)acetates with naphthols and phenols. The proposed coupling reaction proceeded smoothly in the presence of *m*CPBA as an oxidant under ambient conditions to provide arylglycine derivatives in satisfactory yields.

## Supporting Information

File 1 General methods, characterization data and NMR spectra of all synthesized compounds.
