# Supplementary material for: Arylglycine-derivative synthesis via oxidative sp3 C–H functionalization of α-amino esters
Source: Beilstein J Org Chem. 2012 Sep 18;8:1564–8. doi: 10.3762/bjoc.8.178 (PMC3510987; doi:10.3762/bjoc.8.178)
Supplement: File 1 — General methods, characterization data and NMR spectra of all synthesized compounds. [file Beilstein_J_Org_Chem-08-1564-s001.pdf]

Supporting Information

for

Arylglycine-derivative synthesis via oxidative sp^3 C–H functionalization of α-amino esters

Zhanwei Xu, Xiaoqiang Yu*, Xiujuan Feng and Ming Bao*

Address: State Key Laboratory of Fine Chemicals, Dalian University of Technology, Dalian 116023, China

Email: Xiaoqiang Yu - yuxiaoqiang@dlut.edu.cn; Ming Bao - mingbao@dlut.edu.cn

* Corresponding author

General methods, characterization data and NMR spectra of all synthesized compounds

Table of Contents

1. General information ……………………………………………………………….....S2
2. General procedure for the coupling reaction……………………..…….....……........S2
3. Characterization data for all products……….…..…………….…………..………....S2
4. References……….……………….…………………………………………..…..……..S6
5. Copies of ^{1}H and ^{13}C NMR spectra………………….…...……………………….....….S7

1. General information

NMR spectra were run in $CDCl_3$ on a 400 MHz instrument and recorded at the following frequencies: proton (^1H, 400 MHz), carbon (^{13}C, 100 MHz).

2. General procedure for the coupling reaction

To a solution of ethyl 2-(disubstituted amino)acetate (**2**, 0.6 mmol) in CH_2Cl_2 (3.0 mL), *m*CPBA (0.6 mmol, 121.8 mg, 85% purity) and naphthalenol or phenol (**1**, 0.5 mmol) were added. After the resulting mixture had been stirred under ambient conditions for a period (the reaction progress was monitored by TLC), the solvent was removed under reduced pressure. The residue obtained was purified by silica gel chromatography (eluent: petroleum ether/ethyl acetate = 3:1) to afford arylglycine derivatives **3**.

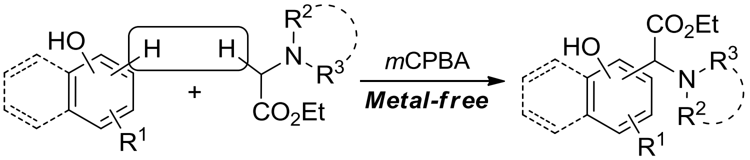

Scheme S1: Oxidative coupling of naphthalenols or phenols with ethyl 2-(disubstituted amino)acetates.

3. Characterization data for all products

Ethyl 2-(2-hydroxynaphthalen-1-yl)-2-morpholinoacetate (3a) [1]

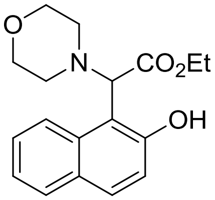

Yield: 79%, 124.5 mg, colorless oil. ^1H NMR ($CDCl_3$, 400 MHz) δ 1.11–1.13 (m, 3H), 2.64 (br, 4H), 3.78–3.82 (m, 4H), 4.02–4.18 (m, 2H), 4.87 (s, 1H), 7.08 (d, J = 8.9 Hz, 1H), 7.29–7.33 (m, 1H), 7.47–7.51 (m, 1H), 7.70–7.75 (m, 2H), 8.03 (d, J = 8.6 Hz, 1H), 11.27 (br, 1H); ^{13}C NMR ($CDCl_3$, 100 MHz) δ 14.1, 51.7, 61.6, 66.8, 108.6, 119.5, 121.7, 123.0, 127.1, 128.9, 129.0, 131.0, 132.7, 156.4, 169.3.

Ethyl 2-(2-hydroxynaphthalen-1-yl)-2-(piperidin-1-yl)acetate (3b)

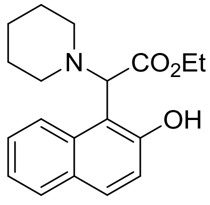

Yield: 64%, 100.3 mg, colorless oil. ^{1}H NMR (CDCl$_3$, 400 MHz) δ 1.11 (t, *J* = 7.1 Hz, 3H), 1.53–1.71 (m, 6H), 2.60 (br, 4H), 4.04–4.13 (m, 2H), 4.83 (s, 1H), 7.09 (d, *J* = 8.9 Hz, 1H), 7.30–7.73 (m, 4H), 8.02 (d, *J* = 8.6 Hz, 1H), 12.03 (br, 1H); ^{13}C NMR (CDCl$_3$, 100 MHz) *δ* 14.1, 24.3, 25.9, 52.5, 61.3, 69.5, 69.6, 109.5, 119.7, 121.7, 122.7, 126.8, 128.7, 128.9, 130.6, 132.7, 157.0, 169.9; IR (neat) 747, 824, 1027, 1117, 1208, 1239, 1301, 1468, 1601, 1623, 1739, 2813, 2855, 2937, 3054 cm^{-1}; HRMS (ES) calcd for $C_{19}H_{23}NO_3Na$: 336.1576 [M + Na]$^+$; found: 336.1592.

Ethyl 2-(benzyl(methyl)amino)-2-(2-hydroxynaphthalen-1-yl)acetate (3c) [1]

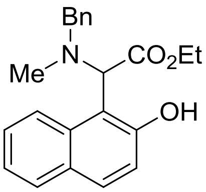

Yield: 64%, 111.8 mg, colorless oil. ^{1}H NMR (CDCl$_3$, 400 MHz) δ 1.13 (t, *J* = 7.1 Hz, 3H), 2.27 (s, 1H), 3.68 (br, 2H), 4.06–4.18 (m, 2H), 4.99 (s, 1H), 7.14 (d, *J* = 8.9 Hz, 1H), 7.27–7.37 (m, 6H), 7.49–7.53 (m, 1H), 7.72–7.77 (m, 2H), 8.06 (d, *J* = 8.6 Hz, 1H), 11.74 (br, 1H); ^{13}C NMR (CDCl$_3$, 100 MHz) δ 14.1, 39.3, 59.6, 61.6, 68.7, 109.8, 119.7, 121.8, 122.9, 127.0, 127.9, 128.7, 128.9, 129.0, 129.7, 130.9, 132.6, 136.4, 156.8, 169.9.

Ethyl 2-(2-hydroxy-4-methylnaphthalen-1-yl)-2-morpholinoacetate (3d)

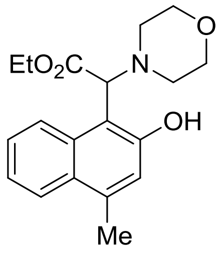

Yield: 79%, 130.1 mg, colorless oil. ^{1}H NMR (CDCl$_3$, 400 MHz) δ 1.12 (t, *J* = 7.1 Hz, 3H), 2.62–2.64 (m, 7H), 3.77–3.81 (m, 4H), 4.01–4.15 (m, 2H), 4.85 (s, 1H), 6.96 (s, 1H), 7.35 (dd, *J* = 7.4, 7.7 Hz, 1H), 7.50 (dd, *J* = 7.5, 7.8 Hz, 1H), 7.91 (d, *J* = 7.5 Hz, 1H), 8.04 (d, *J* = 8.5 Hz, 1H), 11.17 (br, 1H); ^{13}C NMR (CDCl$_3$, 100 MHz) δ 14.2, 19.7, 51.7, 61.6, 66.8,

68.9, 106.8, 120.3, 122.2, 122.8, 125.0, 126.8, 128.3, 133.0, 137.7, 155.8, 169.5; IR (neat) 754, 1030, 1118, 1186, 1267, 1390, 1466, 1604, 1616, 1737, 2854, 2966, 3029 cm^{-1}; HRMS (ES) calcd for $C_{19}H_{23}NO_4Na$: 352.1525 $[M + Na]^+$; found: 352.1520.

Ethyl 2-(2-hydroxy-6-methoxy-4-methylnaphthalen-1-yl)-2-morpholinoacetate (3e)

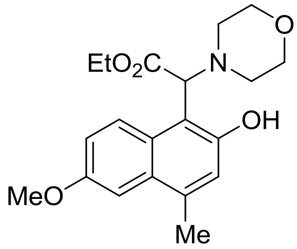

Yield: 75%, 134.8 mg, yellow solid, m.p. 134–136 °C. ^{1}H NMR ($CDCl_3$, 400 MHz) δ 1.12 (t, *J* = 7.1 Hz, 3H), 2.58–2.63 (m, 7H), 3.77–3.84 (m, 4H), 3.92 (s, 1H), 4.03–4.17 (m, 2H), 4.80 (s, 1H), 6.95 (s, 1H), 7.19–7.21 (m, 2H), 7.97 (d, *J* = 10.0 Hz, 1H), 10.92 (br, 1H); ^{13}C NMR ($CDCl_3$, 100 MHz) δ 14.2, 19.9, 51.7, 55.6, 61.6, 66.9, 104.5, 107.1, 118.4, 120.8, 123.8, 128.0, 129.3, 136.2, 154.0, 155.5, 169.5; IR (KBr) 732, 1032, 1118, 1152, 1193, 1237, 1267, 1452, 1525, 1607, 1737, 2854, 2963, 3095 cm^{-1}; HRMS (ES) calcd for $C_{20}H_{25}NO_5Na$: 382.1630 $[M + Na]^+$; found: 382.1624.

Ethyl 2-(1-hydroxynaphthalen-2-yl)-2-morpholinoacetate (3f)

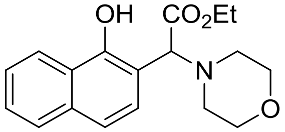

Yield: 66%, 104.1 mg, colorless oil. ^{1}H NMR ($CDCl_3$, 400 MHz) δ 1.18 (t, *J* = 7.1 Hz, 3H), 2.64–2.65 (m, 4H), 3.76–3.86 (m, 4H), 4.07–4.20 (m, 3H), 7.13 (d, *J* = 8.4 Hz, 1H), 7.30 (d, *J* = 8.4 Hz, 1H), 7.43–7.48 (m, 2H), 7.72–7.74 (m, 1H), 8.26–8.29 (m, 1H), 10.83 (br, 1H); ^{13}C NMR ($CDCl_3$, 100 MHz) δ 14.2, 51.6, 61.5, 66.8, 69.4, 114.0, 115.7, 115.8, 123.0, 123.1, 137.7, 139.9, 157.6, 169.6; IR (neat) 799, 888, 1029, 1118, 1152, 1199, 1393, 1453, 1578, 1736, 2854, 2915, 2965, 3055 cm^{-1}; HRMS (ES) calcd for $C_{18}H_{21}NO_4Na$: 338.1368 $[M + Na]^+$; found: 338.1361.

Ethyl 2-(2-hydroxyphenyl)-2-morpholinoacetate (3g)

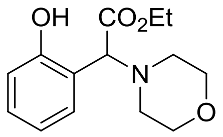

Yield: 30%, 39.8 mg, colorless oil. ^{1}H NMR ($CDCl_3$, 400 MHz) δ 1.21 (t, *J* = 7.1 Hz, 3H), 2.60–2.61 (m, 4H), 3.77–3.79 (m, 4H), 4.08 (s, 1H), 4.11–4.23 (m, 2H), 6.79–6.85 (m, 2H), 7.06 (d, *J* = 7.6 Hz, 1H), 7.19–7.23 (m, 1H), 9.99 (br, 1H); ^{13}C NMR ($CDCl_3$, 100 MHz) δ 14.2, 51.2, 61.6, 66.9, 74.0, 117.1, 118.5, 119.8, 129.7, 130.3, 157.1, 169.6; IR (neat) 755, 885, 1029, 1118, 1154, 1192, 1267, 1488, 1587, 1739, 2854, 2963, 3049 cm^{-1}; HRMS (ES) calcd for $C_{14}H_{19}NO_4Na$: 288.1212 [M + Na]$^+$; found: 288.1219.

Ethyl 2-(5-ethyl-2-hydroxyphenyl)-2-morpholinoacetate (3h)

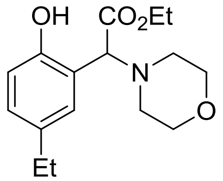

Yield: 30%, 44.1 mg, colorless oil. ^{1}H NMR ($CDCl_3$, 400 MHz) δ 1.16–1.23 (m, 6H), 2.51–2.60 (m, 6H), 3.76–3.78 (m, 4H), 4.04 (s, 1H), 4.14–4.20 (m, 2H), 6.76 (d, *J* = 8.3 Hz, 1H), 6.87 (s, 1H), 7.03 (dd, *J* = 1.6, 8.2 Hz, 1H), 9.77 (br, 1H); ^{13}C NMR ($CDCl_3$, 100 MHz) δ 14.2, 15.9, 28.0, 51.3, 61.5, 66.9, 74.1, 116.8, 118.1, 128.9, 129.5, 135.4, 154.8, 169.7; IR (neat) 830, 889, 1030, 1071, 1212, 1331, 1455, 1501, 1596, 1621, 1739, 2855, 2963, 3171 cm^{-1}; HRMS (ES) calcd for $C_{16}H_{23}NO_4Na$: 316.1525 [M + Na]$^+$; found: 316.1534.

Ethyl 2-(2-hydroxy-4,6-dimethylphenyl)-2-morpholinoacetate (3i)

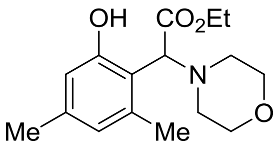

Yield: 35%, 51.4 mg, colorless oil. ^{1}H NMR ($CDCl_3$, 400 MHz) δ 1.20 (t, J = 7.1 Hz, 3H), 2.22 (s, 3H), 2.34 (s, 3H), 2.55 (br, 1H), 3.76–3.78 (m, 4H), 4.09–4.18 (m, 2H), 4.28 (s, 1H), 6.49 (s, 1H), 6.52 (s, 1H), 10.37 (br, 1H); ^{13}C NMR ($CDCl_3$, 100 MHz) δ 14.2, 51.5, 61.6, 67.0, 74.19, 74.23, 111.1, 119.3, 122.78, 122.82, 125.1, 125.3, 126.9, 127.4, 134.6, 153.4, 169.6; IR (neat) 839, 887, 1031, 1118, 1189, 1257, 1319, 1453, 1578, 1625, 1740, 2855, 2916, 2966, 3130 cm^{-1}; HRMS (ES) calcd for $C_{16}H_{23}NO_4Na$: 316.1525 [M + Na]$^+$; found: 316.1519.

Ethyl 2-(2,4-dihydroxyphenyl)-2-morpholinoacetate (3j)

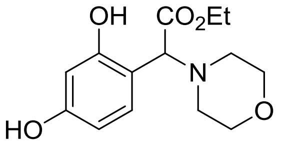

Yield: 55%, 77.4 mg, colorless oil. ^{1}H NMR ($CDCl_3$, 400 MHz) δ 1.21 (t, J = 7.1 Hz, 3H), 2.60 (br, 4H), 3.78 (br, 4H), 4.00 (s, 1H), 4.11–4.23 (m, 2H), 6.29–6.34 (m, 2H), 6.89 (d, J = 8.2 Hz, 1H), 7.94 (br, 1H); ^{13}C NMR ($CDCl_3$, 100 MHz) δ 14.1, 51.2, 61.7, 66.9, 73.4, 103.9, 107.4, 110.5, 130.7, 158.0, 158.1, 170.1; IR (neat) 733, 886, 977, 1029, 1116, 1175, 1268, 1312, 1454, 1516, 1601, 1627, 1732, 2856, 2917, 2968, 3346 cm^{-1}; HRMS (ES) calcd for $C_{14}H_{19}NO_5Na$: 304.1161 $[M + Na]^+$; found: 304.1157.

4. Reference

[1] Hwang, D.-R.; Uang, B.-J. *Org. Lett.* **2002**, *4*, 463–466.

5. Copies of ^{1}H and ^{13}C NMR spectra

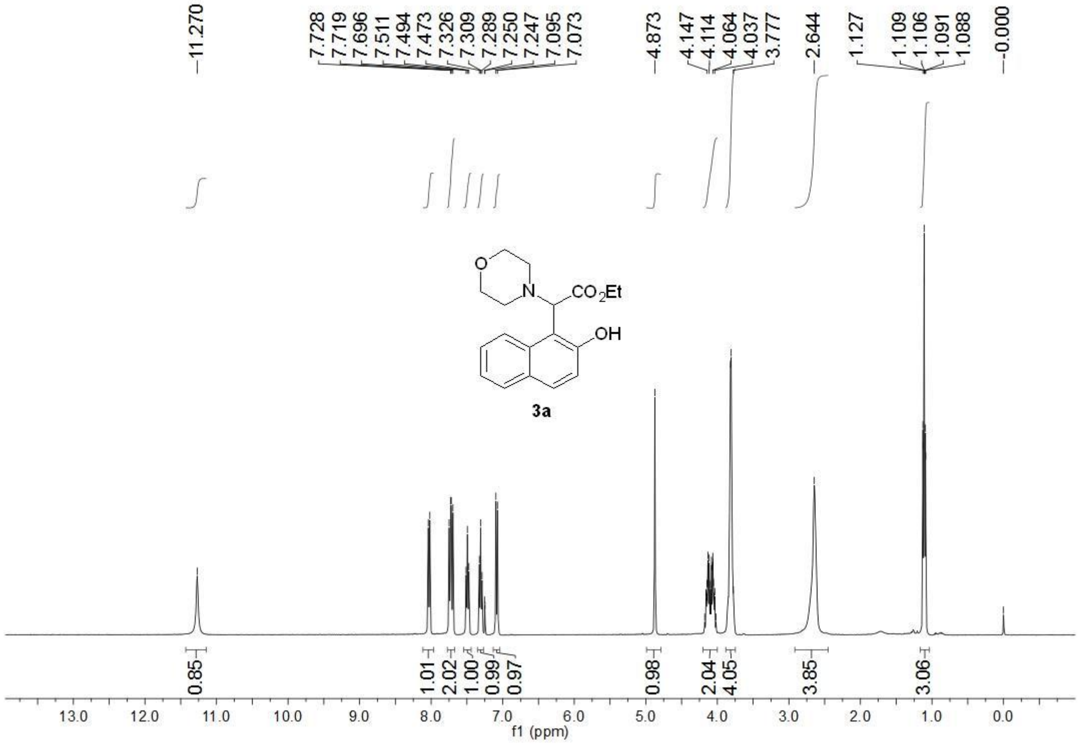

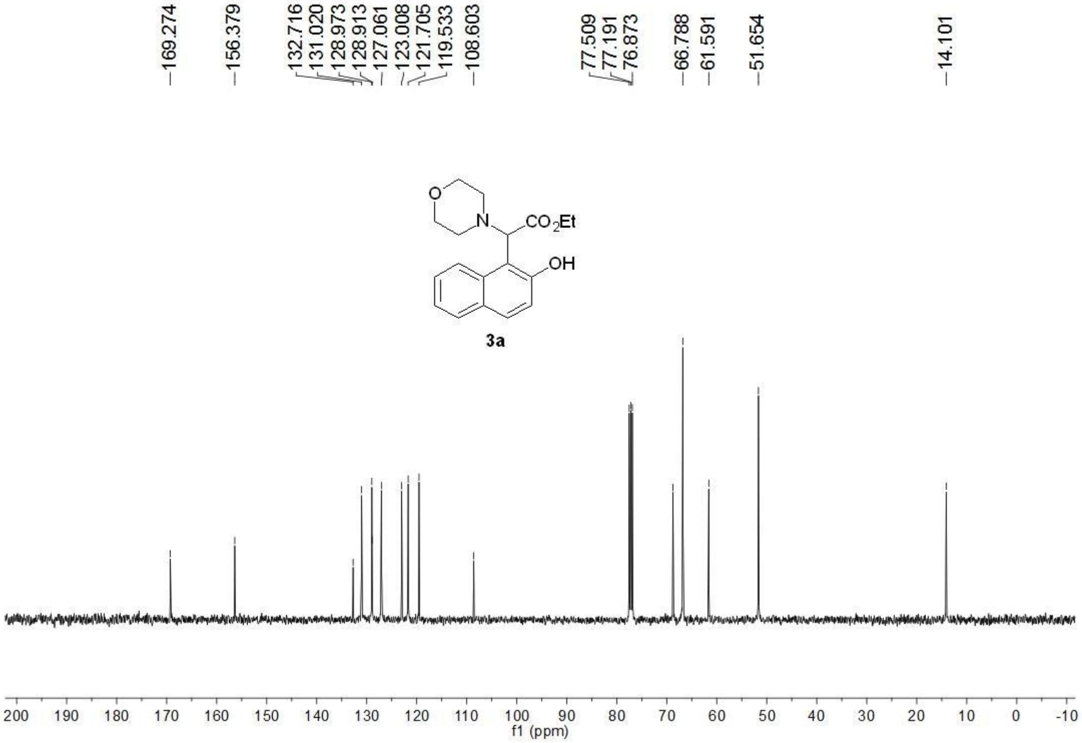

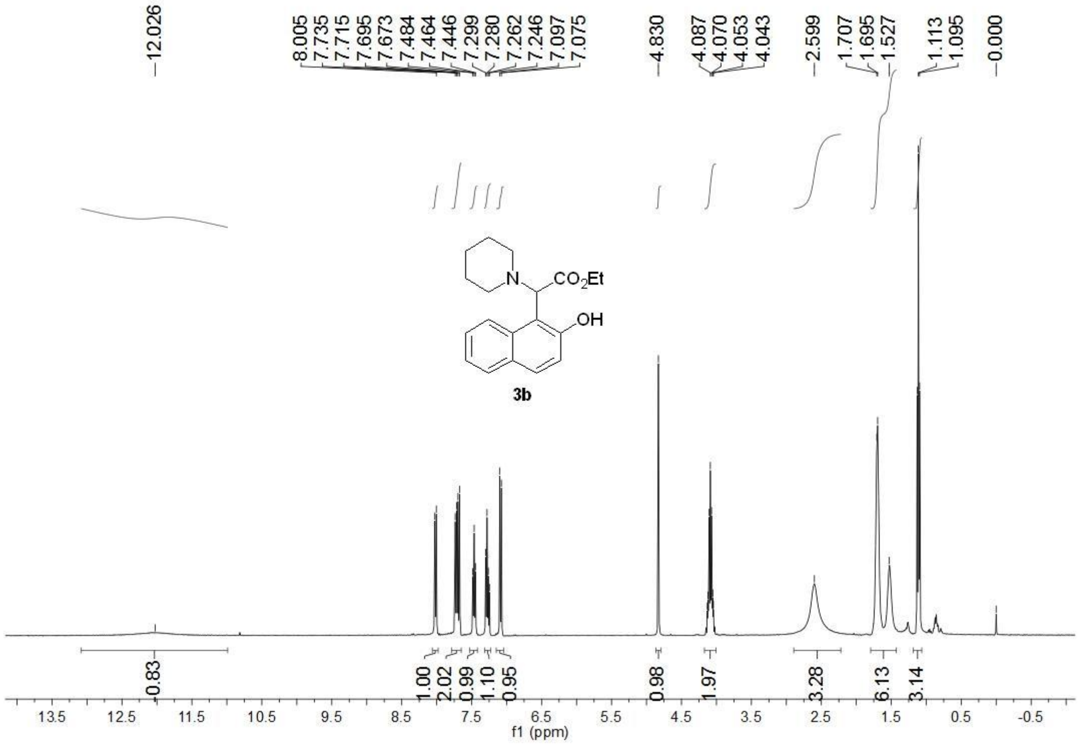
12.026
8.005
7.735
7.715
7.695
7.673
7.484
7.464
7.446
7.299
7.280
7.262
7.246
7.097
7.075
4.830
4.087
4.070
4.053
4.043
2.599
1.707
1.695
1.527
1.113
1.095
0.000
N
CO2Et
OH
3b
0.83
1.00
2.02
0.99
1.10
0.95
0.98
1.97
3.28
6.13
3.14
13.5
12.5
11.5
10.5
9.5
8.5
7.5
6.5
5.5
4.5
3.5
2.5
1.5
0.5
-0.5
f1 (ppm)

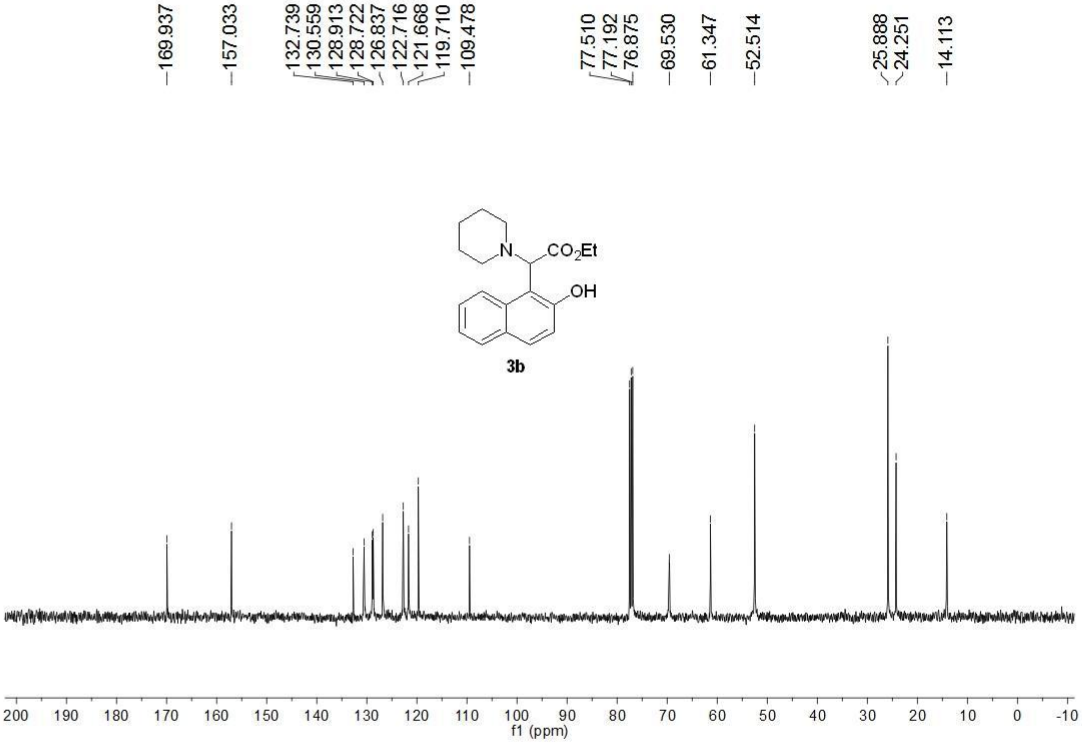
169.937
157.033
132.739
130.559
128.913
128.722
126.837
122.716
121.668
119.710
109.478
77.510
77.192
76.875
69.530
61.347
52.514
25.888
24.251
14.113
N
CO2Et
OH
3b
200
190
180
170
160
150
140
130
120
110
100
90
80
70
60
50
40
30
20
10
0
-10
f1 (ppm)

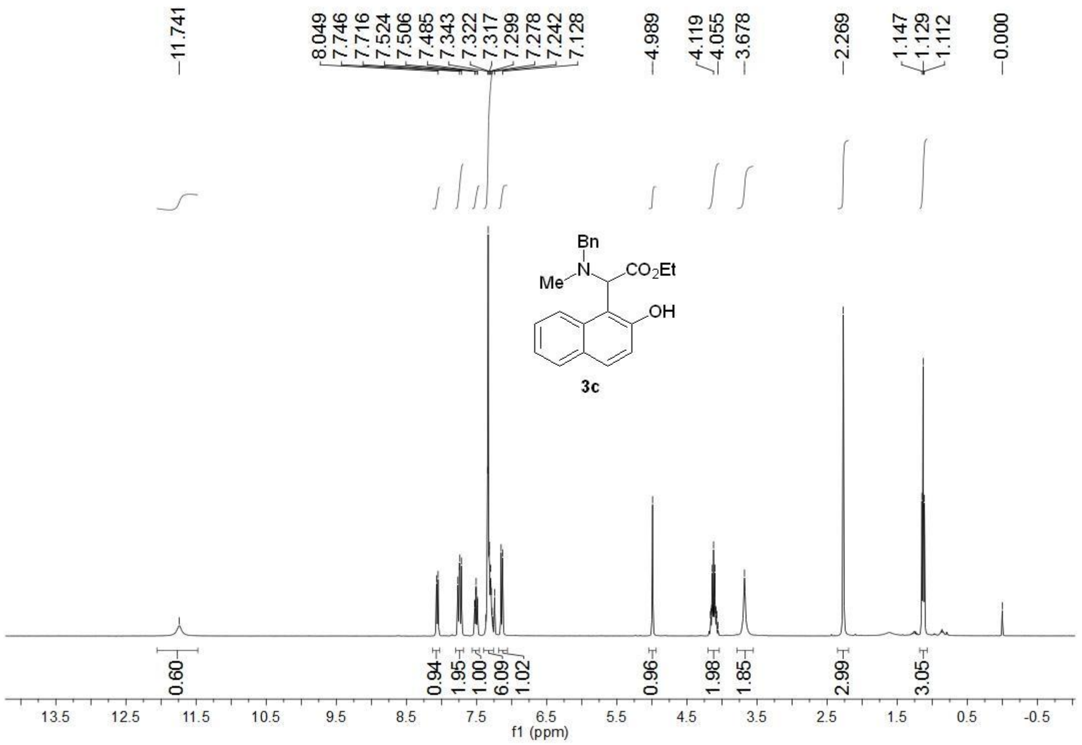
11.741
8.049
7.746
7.716
7.524
7.506
7.485
7.343
7.322
7.317
7.299
7.278
7.242
7.128
4.989
4.119
4.055
3.678
2.269
1.147
1.129
1.112
0.000
Bn
N
Me
CO2Et
OH
3c
0.60
0.94
1.95
1.00
6.09
1.02
0.96
1.98
1.85
2.99
3.05
f1 (ppm)

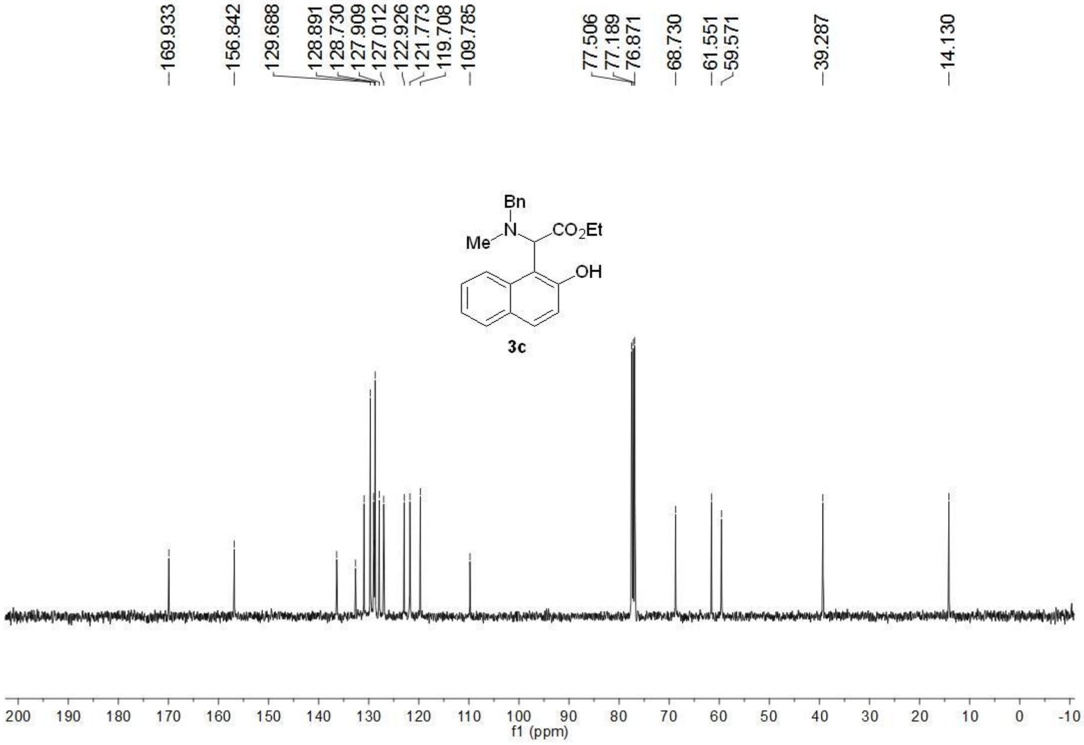
169.933
156.842
129.688
128.891
128.730
127.909
127.012
122.926
121.773
119.708
109.785
77.506
77.189
76.871
68.730
61.551
59.571
39.287
14.130
Bn
N
Me
CO2Et
OH
3c
f1 (ppm)

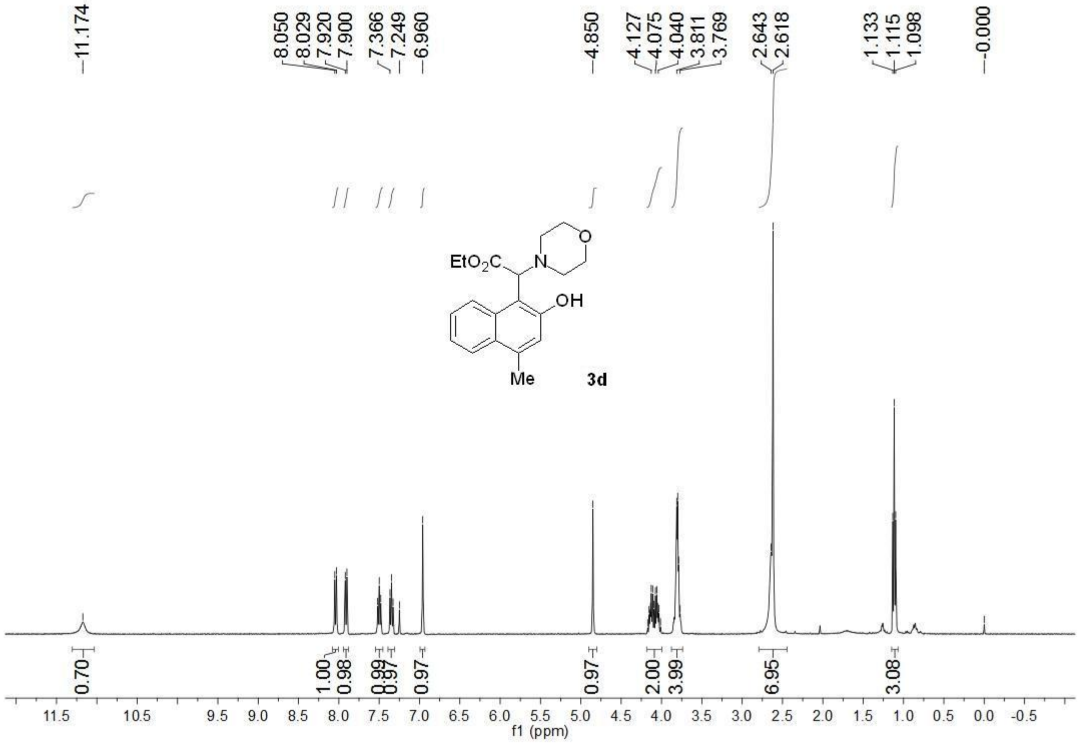
EtO2C
N
O
OH
Me
3d
f1 (ppm)

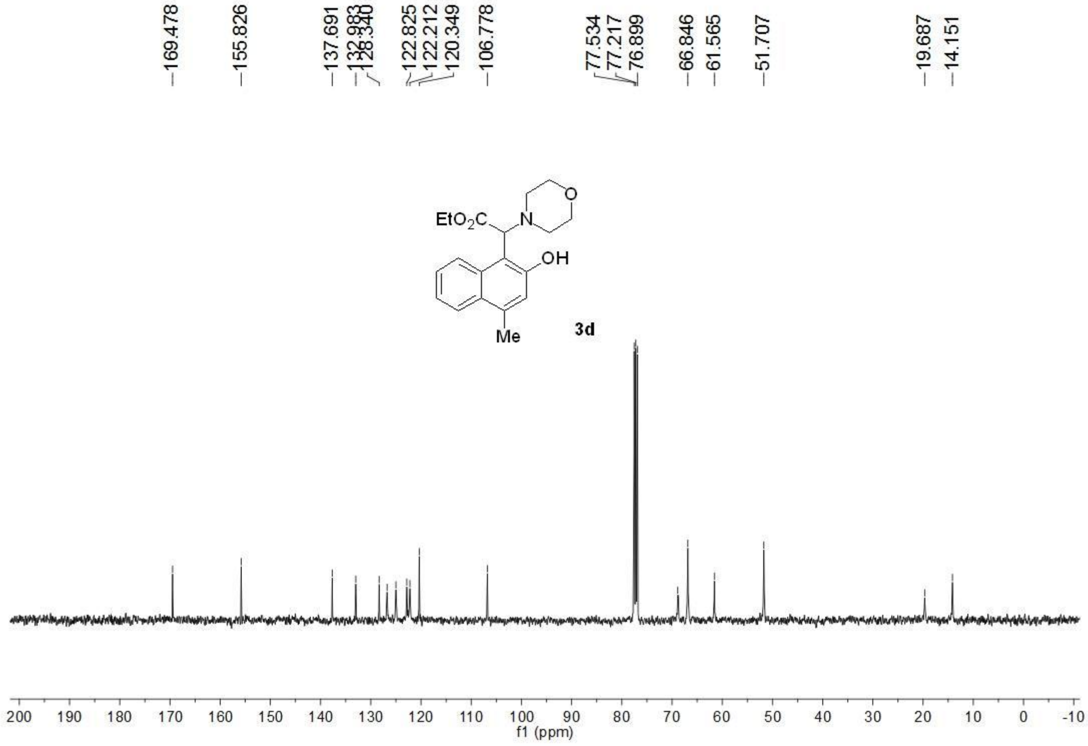
EtO2C
N
O
OH
Me
3d
f1 (ppm)

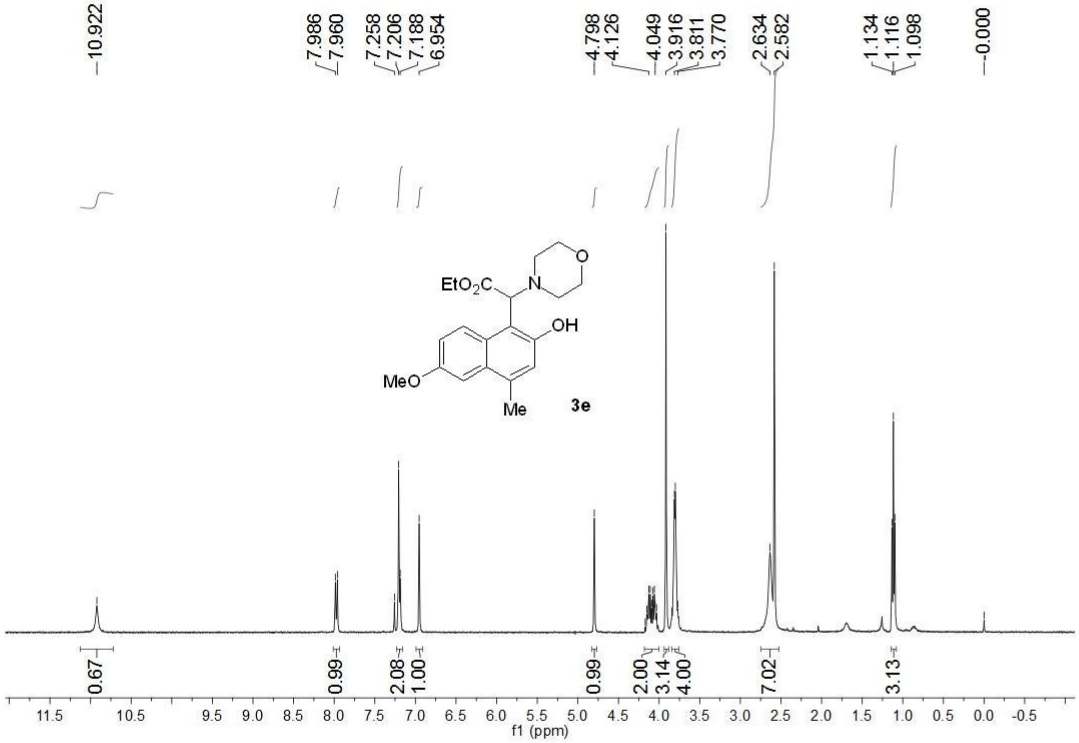
10.922
7.986 7.960 7.258 7.206 7.188 6.954
4.798 4.126 4.049 3.916 3.811 3.770
2.634 2.582
1.134 1.116 1.098
0.000
EtO2C
N
O
OH
MeO
Me
3e
0.67 0.99 2.08 1.00 0.99 2.00 3.14 4.00 7.02 3.13
11.5 10.5 9.5 9.0 8.5 8.0 7.5 7.0 6.5 6.0 5.5 5.0 4.5 4.0 3.5 3.0 2.5 2.0 1.5 1.0 0.5 0.0 -0.5
f1 (ppm)

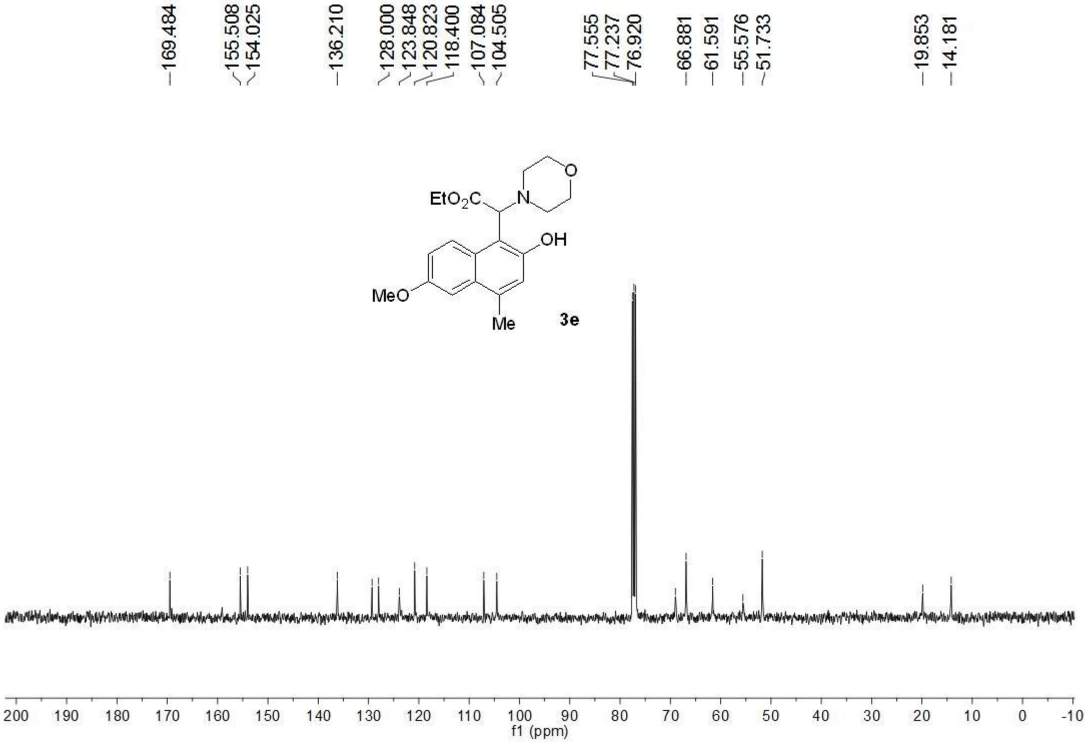
169.484
155.508 154.025
136.210
128.000 123.848 120.823 118.400
107.084 104.505
77.555 77.237 76.920
66.881 61.591 55.576 51.733
19.853 14.181
EtO2C
N
O
OH
MeO
Me
3e
200 190 180 170 160 150 140 130 120 110 100 90 80 70 60 50 40 30 20 10 0 -10
f1 (ppm)

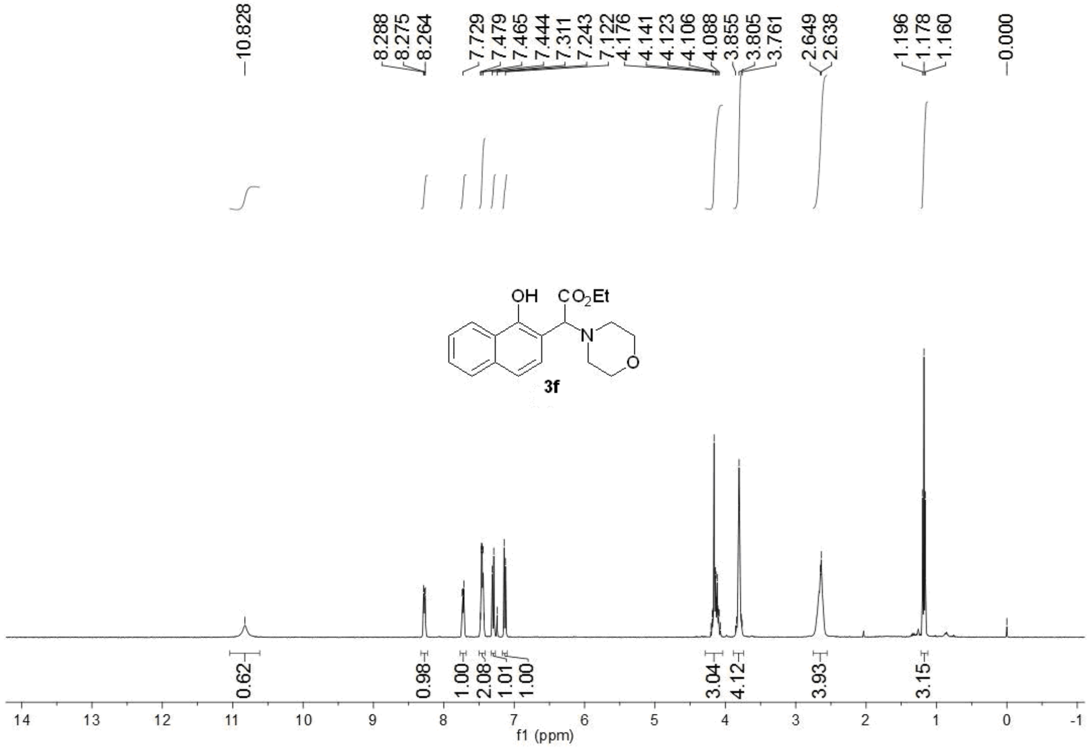
10.828
8.288
8.275
8.264
7.729
7.479
7.465
7.444
7.311
7.243
7.122
4.176
4.141
4.123
4.106
4.088
3.855
3.805
3.761
2.649
2.638
1.196
1.178
1.160
0.000
OH
CO2Et
N
O
3f
0.62
0.98
1.00
2.08
1.01
1.00
3.04
4.12
3.93
3.15
f1 (ppm)

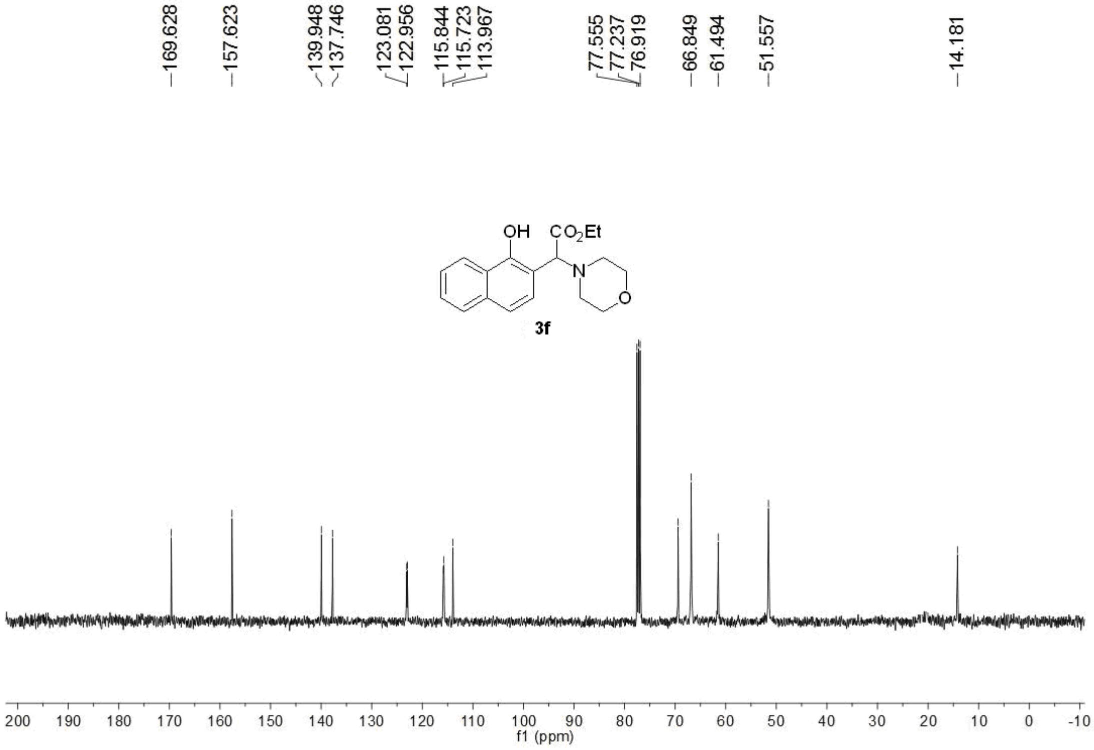
169.628
157.623
139.948
137.746
123.081
122.956
115.844
115.723
113.967
77.555
77.237
76.919
66.849
61.494
51.557
14.181
OH
CO2Et
N
O
3f
f1 (ppm)

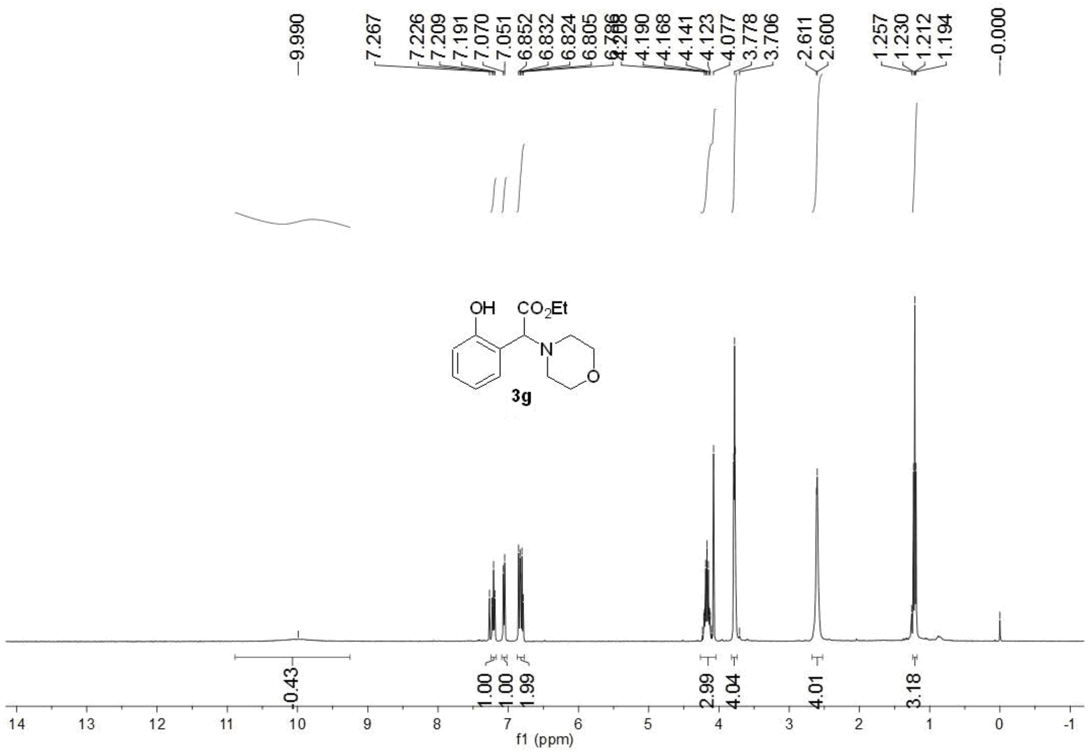

OH
CO2Et
N
O
3g
f1 (ppm)

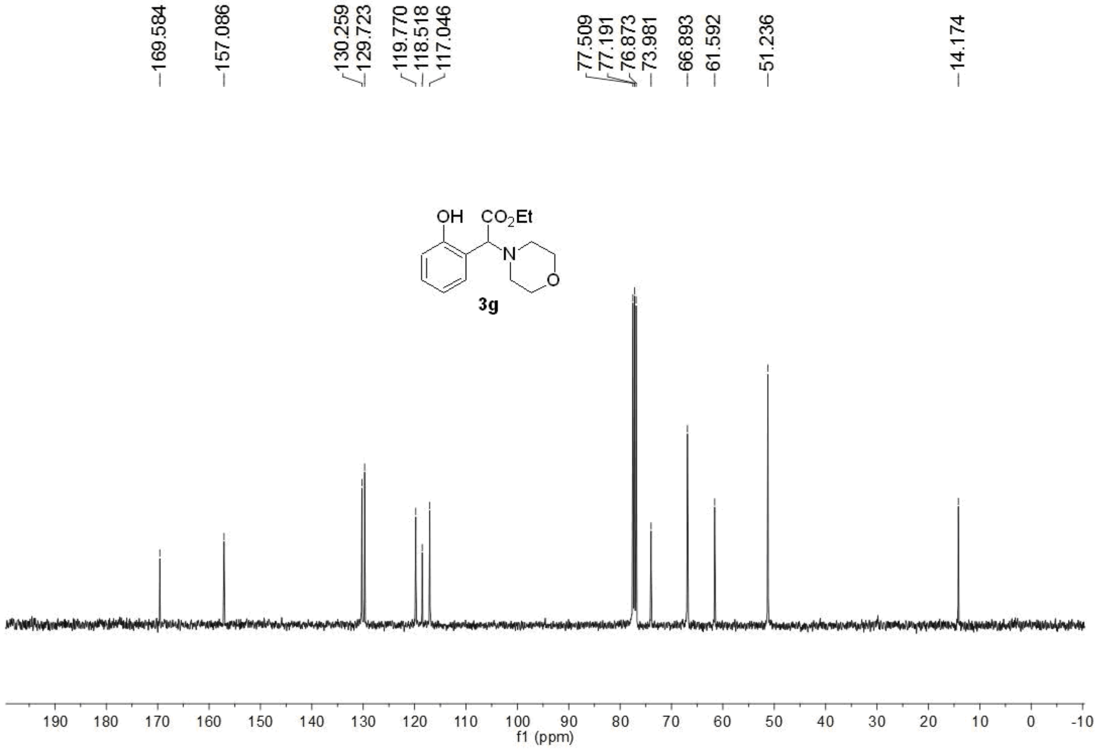

OH
CO2Et
N
O
3g
f1 (ppm)

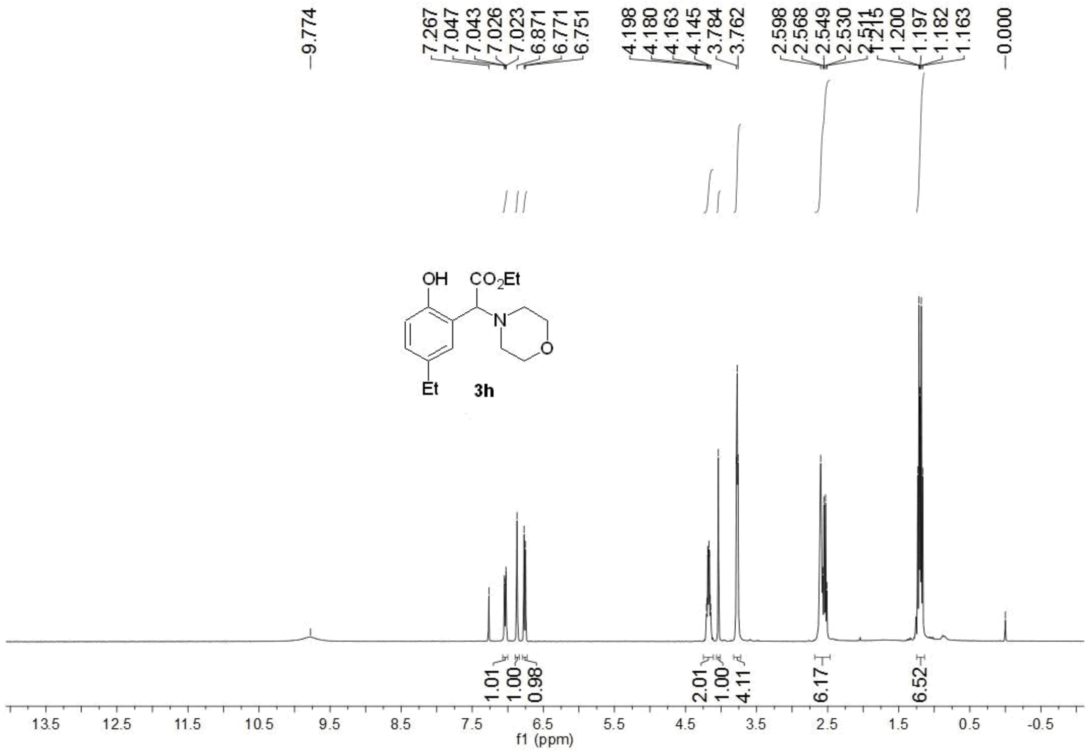
9.774
7.267
7.047
7.043
7.026
7.023
6.871
6.771
6.751
4.198
4.180
4.163
4.145
3.784
3.762
2.598
2.568
2.549
2.530
2.511
1.215
1.200
1.197
1.182
1.163
0.000
OH
CO_2Et
N
O
Et
3h
1.01
1.00
0.98
2.01
1.00
4.11
6.17
6.52
13.5 12.5 11.5 10.5 9.5 8.5 7.5 6.5 5.5 4.5 3.5 2.5 1.5 0.5 -0.5
f1 (ppm)

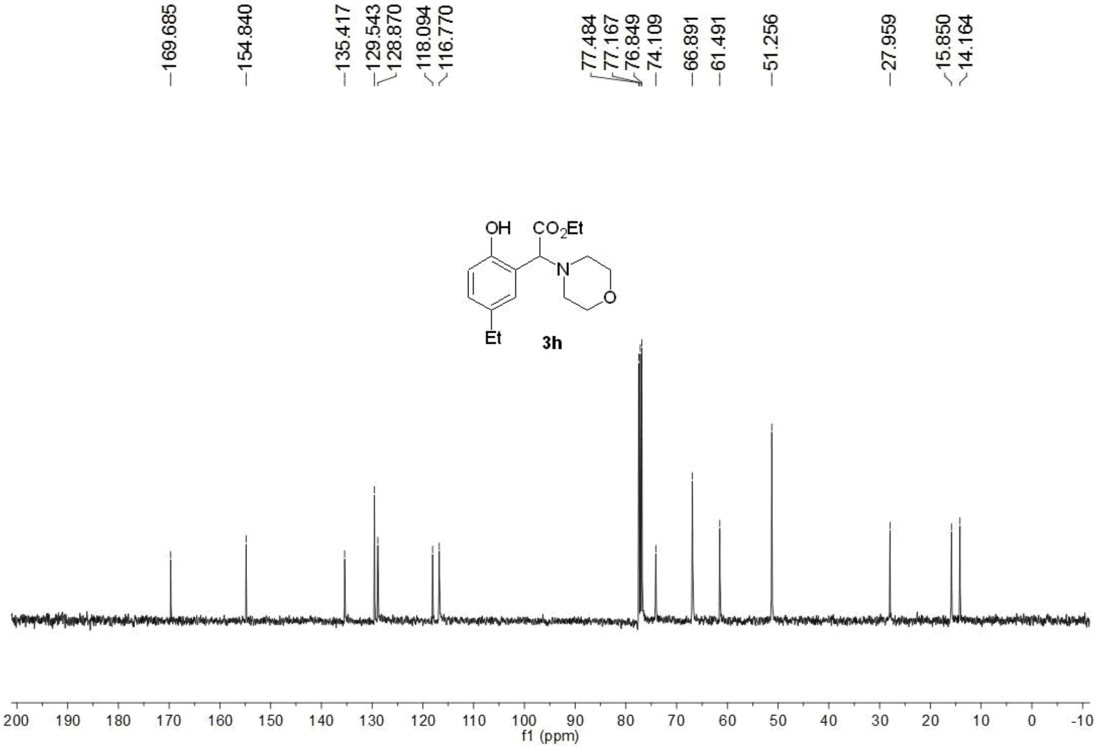
169.685
154.840
135.417
129.543
128.870
118.094
116.770
77.484
77.167
76.849
74.109
66.891
61.491
51.256
27.959
15.850
14.164
OH
CO_2Et
N
O
Et
3h
200 190 180 170 160 150 140 130 120 110 100 90 80 70 60 50 40 30 20 10 0 -10
f1 (ppm)

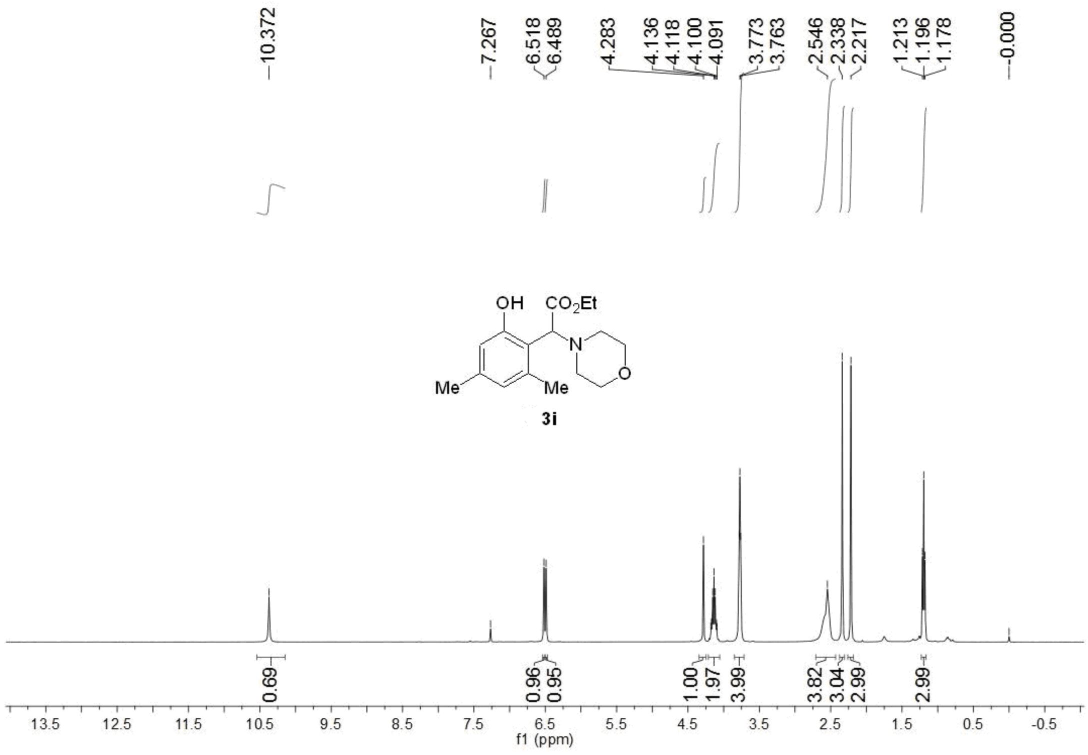
10.372
7.267
6.518
6.489
4.283
4.136
4.118
4.100
4.091
3.773
3.763
2.546
2.338
2.217
1.213
1.196
1.178
0.000
OH
CO_2Et
N
O
Me
Me
3i
0.69
0.96
0.95
1.00
1.97
3.99
3.82
3.04
2.99
2.99
13.5 12.5 11.5 10.5 9.5 8.5 7.5 6.5 5.5 4.5 3.5 2.5 1.5 0.5 -0.5
f1 (ppm)

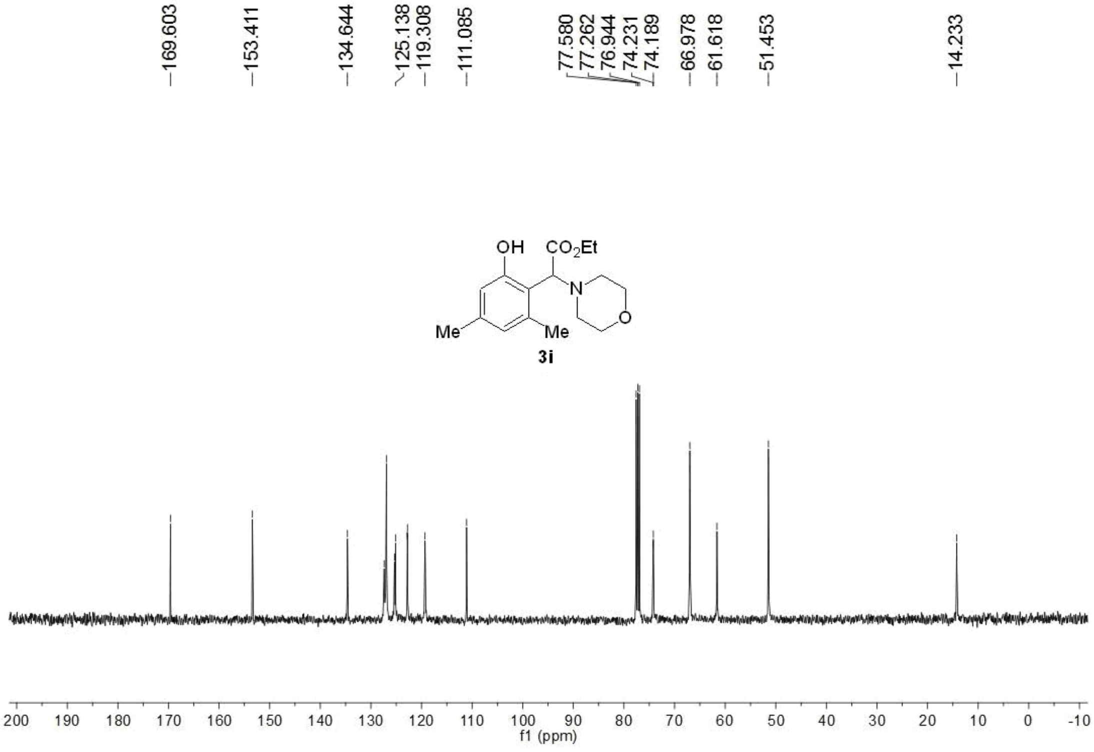
169.603
153.411
134.644
125.138
119.308
111.085
77.580
77.262
76.944
74.231
74.189
66.978
61.618
51.453
14.233
OH
CO_2Et
N
O
Me
Me
3i
200 190 180 170 160 150 140 130 120 110 100 90 80 70 60 50 40 30 20 10 0 -10
f1 (ppm)

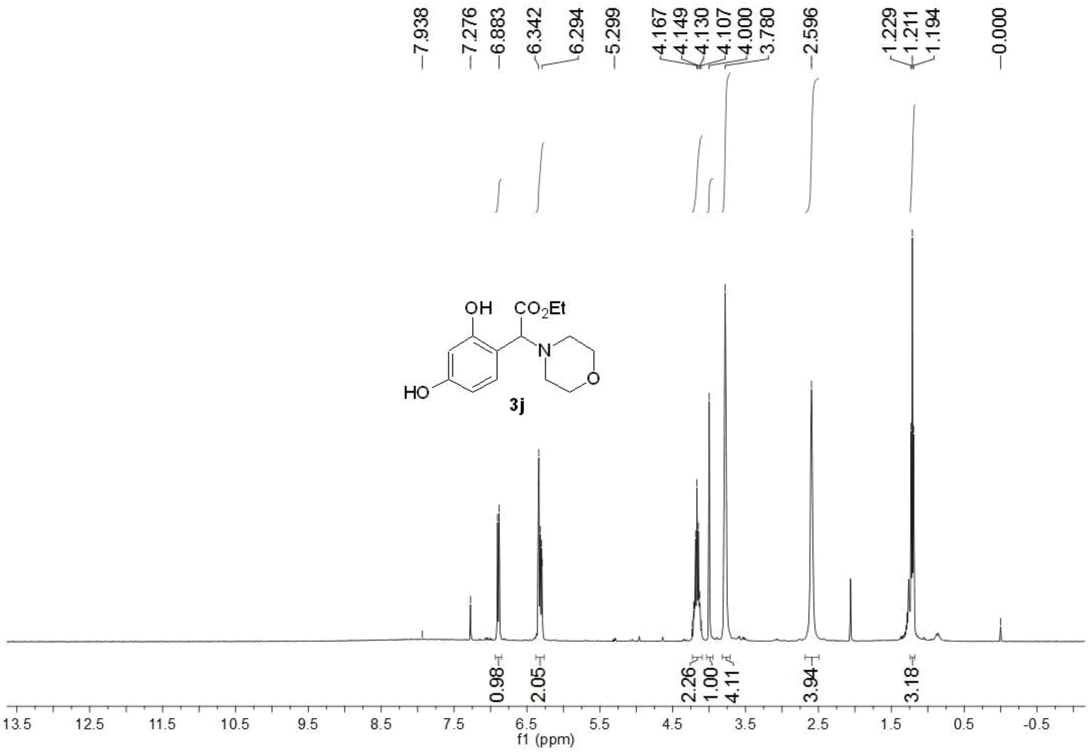
7.938
7.276
6.883
6.342
6.294
5.299
4.167
4.149
4.130
4.107
4.000
3.780
2.596
1.229
1.211
1.194
0.000
OH
CO2Et
N
O
HO
3j
0.98
2.05
2.26
1.00
4.11
3.94
3.18
f1 (ppm)

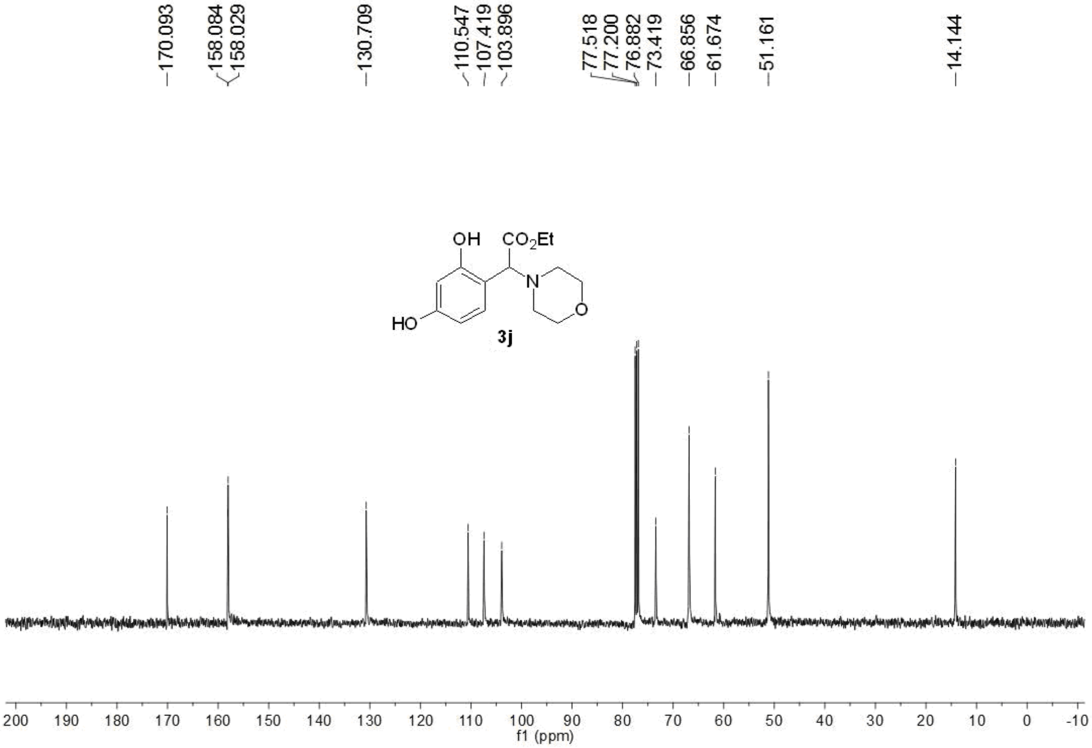
170.093
158.084
158.029
130.709
110.547
107.419
103.896
77.518
77.200
76.882
73.419
66.856
61.674
51.161
14.144
OH
CO2Et
N
O
HO
3j
f1 (ppm)
